# Genetic Variability and Population Structure of the Potential Bioenergy Crop *Miscanthus sinensis* (Poaceae) in Southwest China Based on SRAP Markers

**DOI:** 10.3390/molecules190812881

**Published:** 2014-08-21

**Authors:** Gang Nie, Xin-Quan Zhang, Lin-Kai Huang, Wen-Zhi Xu, Jian-Ping Wang, Yun-Wei Zhang, Xiao Ma, Yan-Hong Yan, Hai-Dong Yan

**Affiliations:** 1 Grassland Science Department, Sichuan Agricultural University, Ya’an 625014, China; E-Mails: nieganggrass@hotmail.com (G.N.); huanglinkai@sicau.edu.cn (L.-K.H.); xuwenzhi_herb@hotmail.com (W.-Z.X.); maroar@126.com (X.M.); yanyanhong3588284@126.com (Y.-H.Y.); yanhaidong1991@163.com (H.-D.Y.); 2 Agronomy Department, University of Florida, Gainesville, FL 32610, USA; E-Mail: wangjp@ufl.edu; 3 Grassland Institute, China Agricultural University, Beijing 100193, China; E-Mail: zywei@126.com

**Keywords:** genetic diversity, population structure, SRAP, *Miscanthus sinensis*

## Abstract

The genus *Miscanthus* has great potential as a biofuel feedstock because of its high biomass, good burning quality, environmental tolerance, and good adaptability to marginal land. In this study, the genetic diversity and the relationship of 24 different natural *Miscanthus sinensis* populations collected from Southwestern China were analyzed by using 33 pairs of Sequence Related Amplified Polymorphism (SRAP) primers. A total of 688 bands were detected with 646 polymorphic bands, an average of 19.58 polymorphic bands per primer pair. The average percentage of polymorphic loci (P), gene diversity (H), and Shannon’s diversity index (I) among the 24 populations are 70.59%, 0.2589, and 0.3836, respectively. The mean value of total gene diversity (*H_T_*) was 0.3373 ± 0.0221, while the allelic diversity within populations (*H_S_*) was 0.2589 ± 0.0136 and the allelic diversity among populations (*D_ST_*) was 0.0784. The mean genetic differentiation coefficient (*Gst* = 0.2326) estimated from the detected 688 loci indicated that there was 76.74% genetic differentiation within the populations, which is consistent with the results from Analysis of Molecular Variance (AMOVA) analysis. Based upon population structure and phylogenetic analysis, five groups were formed and a special population with mixed ancestry was inferred indicating that human-mediated dispersal may have had a significant effect on population structure of *M. sinensis*. Evaluating the genetic structure and genetic diversity at morphological and molecular levels of the wild *M. sinensis* in Southwest China is critical to further utilize the wild *M. sinensis* germplasm in the breeding program. The results in this study will facilitate the biofuel feedstock breeding program and germplasm conservation.

## 1. Introduction

The genus *Miscanthus* is comprised of C_4_ perennial rhizomatous grasses, originated from Eastern Asia. Owing to high biomass productivity [1], low-nutrient input [2,3], and high water-use efficiencies [4], *Miscanthus* have attracted considerable attention as one of the most promising non-food bioenergy crops. There are about 10-15 *Miscanthus* species distributed worldwide, of which seven are native to China [5,6,7,8]. Since the 1970s, this genus, specifically *M. × giganteus*, has been intensively studied in Europe as a biomass feedstock [7,9,10]. However, *M. × giganteus* is propagated by plant rhizomes or tissue culture and does not produce fertile flowers or seeds, and its production is heavily limited by its natural sterility and a narrow genetic base [11]. As a progenitor of *M. × giganteus*, *M. sinensis* is propagated by seeds which is a favorable trait for crop adoption and provides a comparable yield in some places and could be a valuable genetic resource for biofuel crop domestication and improvement [6,12]. Based on the previous tests of drought and cold tolerance of *M. sinensis* in Europe, a much broader range of adaptation than *M. × giganteus* was found in this diploid species [1,11]. So with *M. sinensis* it is considered possible to breed varieties with similar or better yield but higher tolerance for frost and drought than *M. × giganteus.* Besides, it can be used in crosses to create new cultivars of *M. × giganteus*.

Molecular markers are essential tools for germplasm evaluation, genetic analysis, and marker-assisted breeding for crop improvement. Employing molecular markers, such as Sequence Related Amplified Polymorphism (SRAP), Amplified Fragment Length Polymorphism (AFLP), Inter-Simple Sequence Repeats (ISSR) and Simple Sequence Repeats (SSR) [13,14,15,16,17,18,19] to estimate genetic variation within species could assist the breeding program for parental and breeding line selection and desirable traits. SRAP is recognized as a new and useful molecular marker system because of its high reproducibility, low cost, and no requirement of prior knowledge of target sequences [20]. Up to now, SRAP markers have been successfully used for evaluation of genetic diversity for *Carthamus tinctorius*, *Cucurbita pepo*, *buchloe dactyloides*, and *Solanum lycopersicon* [21,22,23,24] and genetic map construction for *Gossypium hirsutum* and *Triticum aestivum* [25,26].

In recent years, many reports on *Miscanthus* were published showing the abundant resources distributed in China [12,27,28,29]. Zhao *et al.* and Clark *et al.* revealed the population structure of *M. sinensis* native to China using SSR and SNP makers respectively. Xu *et al.* used 20 pairs of EST-SSR makers of sorghum to analyze 26 populations of *M. sinensis* from Southwest China, indicating a high genetic diversity and the existence of a gene flow in *M. sinensis* populations in this area. However, the SSR markers used in that study were mainly derived from the non-coding regions, which may not be able to provide sufficient evidence to reveal the diversity and differentiation of *M. sinensis* in Southwest China; therefore, SRAP markers, derived from the coding region, were used in this study. Southwest China, as one of the 34 biodiversity hot spots around the World, has abundant wildlife resources [30]. It is crucial to evaluate the genetic structure and genetic diversity of the wild *M. sinensis* germplasm, which is widely distributed in Southwest China and to eventually utilize this valuable germplasm for crop improvement. However, there are no thorough studies on the genetic diversity and population structure of the germplasm distributed in Southwest China. Therefore, in this study, we evaluated the genetic diversity and population structure of 24 *M. sinensis* natural populations collected in Southwest China using SRAP markers to facilitate the conservation of the *Miscanthus* germplasm and breeding in the near future.

## 2. Results and Discussion

### 2.1. Polymorphism of SRAP Markers

Six accessions of *M. sinensis*, which have significant differences among the morphological characterization and geographic location, were selected to screen 100 pairs of SRAP primers. In total, 33 of them generated robust discernible bands (Table 1). These 33 SRAP primer pairs were then used to genotype the whole collection of 260 individuals. In total, 688 bands were generated and 646 (93.90%) were polymorphic. The number of bands per primer pairs ranged from 13 to 30, with an average of 20.58 bands, of which 19.58 in average were polymorphic. Primer pairs Me6 + em10 amplified the most number of polymorphic bands (30) while Me7 + em1 amplified the least number of polymorphic bands (9). The polymorphic information content (PIC) values ranged from 0.23 (Me3 + em5) to 0.41 (Me4 + em10) with a mean of 0.34, demonstrating a good discriminatory capacity (Table 1).

### 2.2. Genetic Diversity and AMOVA Analysis

The 24 wild distribution populations were comprised of 260 individuals which had a varied genetic diversity reflected by the three main genetic diversity parameters including percentage of polymorphic bands (P), Nei’s [31] gene diversity (H), and Shannon’s Information Index of Diversity (I). Among the 24 populations, the P value ranged from 21.22% (Pop12) to 84.30% (Pop15), with an average of 70.59%. The H value ranged from 0.0787 (Pop12) to 0.3052 (Pop15), with an average of 0.2589 at the population level. The variation trend of the I value was similar to the other two parameters, with an average of 0.3836 (Table 2). The total numbers of P, H and I were 93.90%, 0.3377 and 0.5032 within species, respectively. The genetic data exhibited a high level of genetic diversity within *M. sinensis* species from southwest China.

**Table 1:** Primer sequences amplification information of the SRAP markers.

Pirmer Pairs	Sequence 5'→3'	Total Number of Bands	Number of Polymorphic Bands	Percentage of Polymorphic Bands (%)	Polymorphic Information Content (PIC)
Me1 + em3	TGAGTCCAAACCGGATA GACTGCGTACGAATTGAC	15	15	100.00	0.33
Me1 + em8	TGAGTCCAAACCGGATA GACTGCGTACGAATTCTG	16	13	81.25	0.34
Me1 + em10	TGAGTCCAAACCGGATA GACTGCGTACGAATTCAG	21	21	100.00	0.38
Me2 + em1	GACTGCGTACGAATTTGC GACTGCGTACGAATTAAT	21	21	100.00	0.39
Me2 + em9	GACTGCGTACGAATTTGC GACTGCGTACGAATTCGA	17	14	82.35	0.28
Me2 + em10	GACTGCGTACGAATTTGC GACTGCGTACGAATTCAG	29	28	96.55	0.40
Me3 + em5	TGAGTCCAAACCGGAAT GACTGCGTACGAATTACC	18	15	83.33	0.23
Me3 + em9	TGAGTCCAAACCGGAAT GACTGCGTACGAATTCGA	18	17	94.44	0.31
Me3 + em10	TGAGTCCAAACCGGAAT GACTGCGTACGAATTCAG	24	24	100.00	0.34
Me4 + em1	TGAGTCCAAACCGGACC GACTGCGTACGAATTAAT	16	16	100.00	0.28
Me4 + em7	TGAGTCCAAACCGGACC GACTGCGTACGAATTCAA	22	21	95.45	0.35
Me4 + em9	TGAGTCCAAACCGGACC GACTGCGTACGAATTCGA	20	18	90.00	0.29
Me4 + em10	TGAGTCCAAACCGGACC GACTGCGTACGAATTCAG	20	19	95.00	0.41
Me5 + em2	TGAGTCCAAACCGGAAG GACTGCGTACGAATTTGC	21	19	90.48	0.30
Me5 + em4	TGAGTCCAAACCGGAAG GACTGCGTACGAATTTGA	19	17	89.47	0.31
Me5 + em8	TGAGTCCAAACCGGAAG GACTGCGTACGAATTCTG	25	25	100.00	0.38
Me5 + em10	TGAGTCCAAACCGGAAG GACTGCGTACGAATTCAG	23	23	100.00	0.33
Me6 + em7	TGAGTCCAAACCGGTAA GACTGCGTACGAATTCAA	18	17	94.44	0.34
Me6 + em8	TGAGTCCAAACCGGTAA GACTGCGTACGAATTCTG	27	23	85.19	0.25
Me6 + em10	TGAGTCCAAACCGGTAA GACTGCGTACGAATTCAG	30	30	100.00	0.38
Me7 + em1	TGAGTCCAAACCGGTCC GACTGCGTACGAATTAAT	13	9	69.23	0.28
Me7 + em5	TGAGTCCAAACCGGTCC GACTGCGTACGAATTACC	24	22	91.67	0.33
Me7 + em8	TGAGTCCAAACCGGTCC GACTGCGTACGAATTCTG	16	15	93.75	0.33
Me7 + em10	TGAGTCCAAACCGGTCC GACTGCGTACGAATTCAG	24	23	95.83	0.39
Me8 + em5	TGAGTCCAAACCGGTGC GACTGCGTACGAATTACC	22	22	100.00	0.37
Me8 + em7	TGAGTCCAAACCGGTGC GACTGCGTACGAATTCAA	19	18	94.74	0.40
Me8 + em9	TGAGTCCAAACCGGTGC GACTGCGTACGAATTCGA	23	23	100.00	0.32
Me9 + em1	TGAGTCCAAACCGGTAG GACTGCGTACGAATTAAT	25	24	96.00	0.33
Me9 + em5	TGAGTCCAAACCGGTAG GACTGCGTACGAATTACC	18	16	88.89	0.36
Me9 + em8	TGAGTCCAAACCGGTAG GACTGCGTACGAATTCTG	17	15	88.24	0.34
Me9 + em9	TGAGTCCAAACCGGTAG GACTGCGTACGAATTCGA	21	19	90.48	0.36
Me10 + em1	TGAGTCCAAACCGGTTG GACTGCGTACGAATTAAT	22	21	95.45	0.38
Me10 + em2	TGAGTCCAAACCGGTTG GACTGCGTACGAATTTGC	24	23	95.83	0.38
Total		688	646		
Mean		20.85	19.58	93.27	0.34

**Table 2:** Genetic diversity of *M. sinensis* wild populations.

Population Identity	Sample Size	H	I	P
Pop1	15	0.2712	0.4062	77.91%
Pop2	13	0.2939	0.4351	79.80%
Pop3	12	0.2877	0.4261	77.76%
Pop4	13	0.2852	0.4237	78.78%
Pop5	11	0.2727	0.4042	73.98%
Pop6	8	0.2599	0.3830	68.46%
Pop7	11	0.2849	0.4206	76.60%
Pop8	10	0.2792	0.4127	74.71%
Pop9	8	0.2734	0.4035	73.11%
Pop10	12	0.2799	0.4130	75.00%
Pop11	9	0.2580	0.3819	70.06%
Pop12	5	0.0787	0.1162	21.22%
Pop13	15	0.2773	0.4122	77.91%
Pop14	5	0.2480	0.3624	62.79%
Pop15	18	0.3052	0.4540	84.30%
Pop16	14	0.2828	0.4209	79.22%
Pop17	16	0.2591	0.3910	77.91%
Pop18	11	0.2716	0.4052	76.45%
Pop19	8	0.2679	0.3930	69.04%
Pop20	15	0.2731	0.4090	78.49%
Pop21	12	0.2708	0.4024	75.00%
Pop22	6	0.2360	0.3484	62.65%
Pop23	6	0.2114	0.3116	55.81%
Pop24	7	0.1847	0.2700	47.24%
Mean		0.2589	0.3836	70.59%
Within Species	260	0.3377	0.5032	93.90%

Note: H, Nei’s gene diversity; P, Percentage of Polymorphic Bands; I, Shannon’s Information Index of Diversity.

The total gene diversity (H_T_) was 0.3373 ± 0.0221, while the gene diversity within populations (H_S_) was 0.2589 ± 0.0136 and the gene diversity among populations (D_ST_) were 0.0784. The mean genetic differentiation coefficient (G_ST_ = 0.2326) estimated from the 688 bands indicated that there were 76.74% genetic variation within populations. These results demonstrated that the accessions had a higher level of genetic variation within populations than among them. The AMOVA analysis (Table 3) of the *M. sinensis* wild populations showed similar results that both the genetic variations within (86.0%) and among (14.0) populations were significant. In addition, there was a high frequency of gene flow (Nm = 1.6493) between populations, indicating there were more than one effective immigrants from one population into another at each generation.

**Table 3:** AMOVA analysis of variance distribution with and amoung *M. sinensis* wild populations.

Source	Degree of Freedom	Sum of Squares	Summary of Matches	Percentage of Variation
Among Pops	23	6468.464	281.238	14%
Within Pops	236	24162.059	102.382	86%
Total	259	30630.523		100%

*Miscanthus* is widely distributed around the world, although its main distribution area or diversity center is in China [18,32]. Knowing the relationship and population structure of *M. sinensis* is important for their conservation and utilization [15]. In this study, the high level of genetic diversity of *M. sinensis* from southwest China was revealed by SRAP markers, which are similar to the previous results with EST-SSR, SSR and AFLP markers [9,12,18]. Meanwhile, SRAP analysis indicated higher genetic variation (76.74%) existed within populations than among populations, which is in agreement with the results in other grass species assessed by allozymes, ISSR, RAPD, SSR, and EST-SSR [12,33,34,35,36,37,38] and in *M. sinensis* of China assessed by SNP and SSR makers [18,32]. The main factors determining the plant population genetic structure include the mating and reproduction system, selection pressure, adaptation, and geographic locations [39]. The genetic recombination promotes genetic diversity within populations [40]. In plants, gene flow events can be initiated through pollen or seed. *M. sinensis* is an out-crossing species that can lead to a strong gene flow (Nm = 1.6493) and introgression among populations, so it is reasonable that the genetic variation within populations is greater than that among populations [41,42].

### 2.3. Population Structure and Cluster Analysis

**Figure 1:**
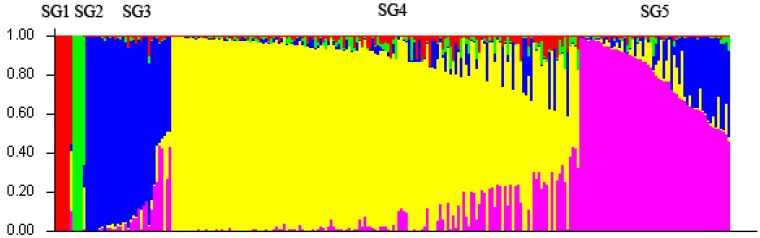
Five subgroups of 260 *M. sinensis* accessions inferred from STRUCTURE analysis. The vertical coordinate of each subgroup indicates the membership coefficients for each individual. Red zone: SG1; Green zone: SG2; Blue zone: SG3; Yellow zone: SG4; Pink zone: SG5.

The population structure of the 260 individuals was estimated under the Hardy-Weinberg Equilibrium by using STRUCTURE V2.3.3 software. Based on maximum likelihood and delta K (∆K) values, the number of optimum subgroups was five (Figure 1). By using a membership probability threshold (Q) of 0.60, majority of the individuals were clearly assigned to specific groups. Among them, 6 individuals were assigned to subgroup (SG) 1 with the accessions mainly collected from Pop24; 5 individuals to SG2 with the accessions mainly collected from Pop12; 27 individuals to SG3 with the accessions mainly collected from Pop13 and Pop15; 150 individuals to SG4 with the accessions mainly collected from Pop2, Pop3, Pop4, Pop5, Pop6, Pop7, Pop8, Pop9, Pop10, Pop11, Pop14, Pop18, Pop19, Pop21, Pop22, Pop23; 48 individuals to SG5 with the accessions mainly collected from Pop1, Pop16, Pop17 and Pop20; 24 individuals were retained in the admixed group (Table S1).

The genetic similarities (GS) of 260 individuals ranged from 0.565 to 0.972 with an average of 0.659 which showed a high level of genetic variation range among the accessions. The Un-weighted Pair-group Method with Arithmetic mean (UPGMA) dendrogram based on GS data obviously revealed that when at the genetic similarity coefficient value of 0.659, five major clusters were formed and group 1 accessions were mainly collected from the Yuxi area of Yunnan. The genotypes of group 2 were primarily collected from Zigong and Jian’ge in Sichuan. Group 3 contained mostly accessions from Jiangyou and Guangyuan. Group 4 accessions were mainly collected from Yaan, Daying, Banan and Zunyi (Figure S1). The rest of the accessions assigned to group 5. The results from the cluster analysis were similar with those from the structure analysis.

**Figure 2:**
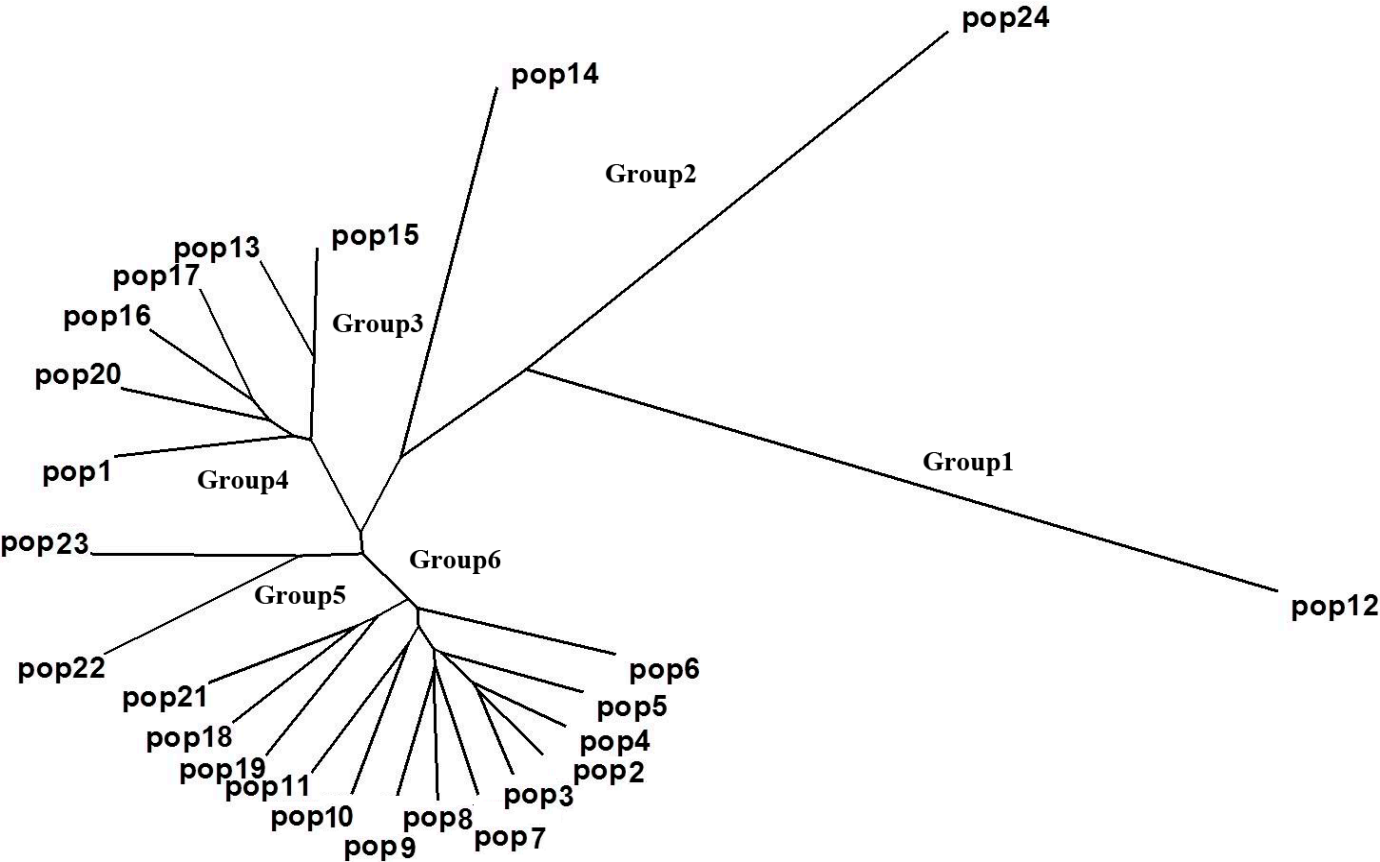
Dendrogram of 24 *M. sinensis* populations based on GD data by UPGMA cluster analysis.

The genetic distances (GD) among the 24 populations were estimated by Nei’s [43] unbiased measure, which could obviously reveal the genetic relationship. The GD between Pop2 from Bifengxia and Pop3 from Baoxing was the lowest (0.028), and the distance between Pop12 from Zigong and Pop24 from Yuxi was the highest (0.292) with the mean of 0.097 (Table S2). The UPGMA dendrogram based on GD data clearly showed the relationships among the 24 populations (Figure 2), which was nearly congruent with the previous cluster analysis of 260 individuals. However, in this result, we found that a new group (Group 5) including Pop22 and Pop23 collected from Guizhou. Throughout the results of the two methods at different levels, we found that combining the analysis is the best strategy to reveal the genetic structure of *M. sinensis* in Southwestern China.

Apparently, Pop12 and Pop24 were differentiated from the other populations in both Structure and UPGMA analysis. The main reason could be the distinct geographic isolation between this two and the rest populations. In addition, Pop12 has the lowest genetic diversity parameters which are P (21.22%), H (0.0787), and I (0.1162), and a low gene flow exists between Pop12 and other populations. Furthermore, Pop12 has a narrow distribution range in this area and almost no other *M. sinensis* plants were found within a range of 5 km around it. Therefore, a habitat fragmentation was formed as influenced by founder effect.

Through the structure analysis, 14 out of 260 individuals with mixed ancestry were all from Pop16. In principle, all of the genetic material of the sampled individuals comes from one or more of K unobserved populations with each population characterized by a set of allele frequencies at each locus. When individuals have mixed ancestry, this means that each genotyped allele comes from one or more populations. We synthesized geographic information to analysis the accessions from Pop16 and found that they all collected along with the G42 highway in China which is the only entrance to the Dead Sea of China located in Da Ying County. The Dead Sea of China is a famous scenic spot where the total number of tourists is approximately 3 million per year. The huge traffic flow and the complex environment could help the seed spread widely. Hence a high gene flow occurred in this area and the plants there had a mixed ancestry. Although some researchers think these man-made factors contribute to the long term survival of populations, this is controversial [44], as several studies [45,46,47,48] indicate that they should not be neglected because those factors accelerate the loss of genetic variability through random genetic drift [49].

The previous clustering result of *M. sinensis* from southwest China assessed using SSR makers [12] was different from the dendrogram that resulted from SRAP makers. These differences could be due to the different DNA segment targets of SSR and SRAP makers. The SSR have a random distribution within the genome, while the target locus of SRAP is mainly in open reading frame regions [20,50]. SSRs mostly exists in non-genic regions, could be in genic regions as well, but with low frequency [50,51]. In different plant individuals, the number of repeat units varies, but the flanking sequence is conserved around the SSR. The numbers of loci studied and their coverage of the genome wide are important in obtaining reliable estimates of genetic relationships between populations and within population [52]. Although, both SRAP and SSR distinguished intraspecific taxa with similar great discriminating power, the average numbers of bands generated by each primer pair of SSR (14.80) [12] were much lower than that of SRAP (20.85). Therefore, we considered that SRAP was more efficient than SSR for assessing the genetic diversity of large numbers of *M. sinensis* accessions. In total, as widely used PCR-based markers, SRAP has advantages over SSR markers, since no prior knowledge of target sequences is required which make it to be widely utilized.

## 3. Experimental Section

### 3.1. Plant Material Collection

The experimental materials consisted of 260 individuals of *M. sinensis* collected from 24 natural populations in Sichuan, Chongqing, Guizhou, and Yunnan provinces in 2010 (Table 4).

**Figure 3:**
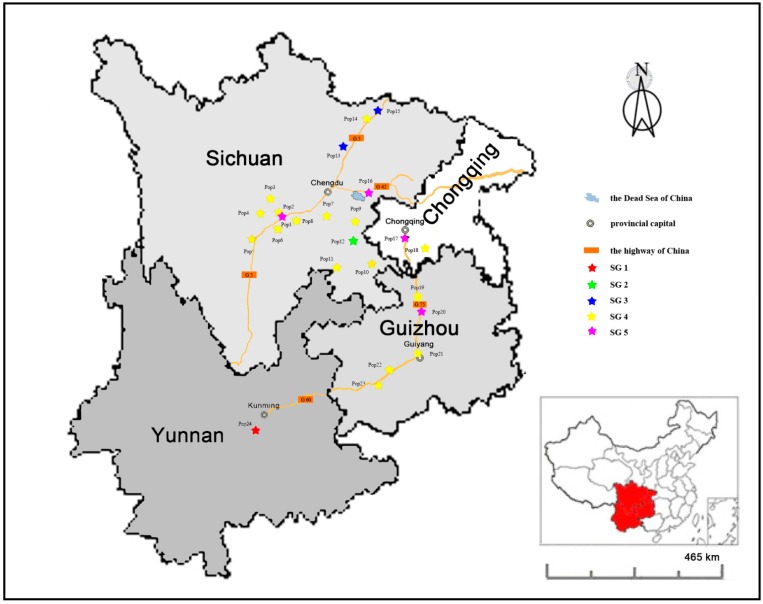
The geographical distribution of 24 populations of *M. sinensis* used in this study. The accessions were mainly sampled from four provinces, Sichuan, Chongqing, Guizhou and Yunnan in China. The different colors pentagram represents the five subgroups generated by STRUCTURE V2.3.3 software.

The sampling locations were selected according to *M. sinensis* habitats based on geographic location and topography. All of approaches used in collecting samples are based on Xu’s method [12] (Figure 3). Within each population, the numbers of appropriate representative individuals were selected based on the size of each population.

**Table 4:** Geographic information of the 24 populations of *M. sinensis*.

Population Identity	Sample Size	Region	Altitude (m)	Latitude (N)	Longitude (E)	Habital
Pop1	15	Ya’an	677	29°58'39.3''	102°59'24.5''	Glade at hillside
Pop2	13	Bi Feng Xia	989	30°07'25.5''	102°59'48.6''	Highway side
Pop3	12	Bao Xing	1253	30°16'05.7''	102°49'19.5''	River beach
Pop4	13	Erlang Mountain	2091	29°51'19.8''	102°18'58.3''	Glade at hillside
Pop5	11	Tuowu Mountain	1630	28°59'52.9''	102°18'12.9''	Glade at hillside
Pop6	8	Niba Mountain	1636	29°39'46.1''	102°36'25.0''	Glade at hillside
Pop7	11	Renshou	471	30°01'05.9''	103°58'21.8''	Hillside
Pop8	10	Hongya	493	29°51'22.6''	103°14'03.5''	Dam
Pop9	8	Zizhong	350	29°48'50.4''	104°42'28.6''	Glade in orangery
Pop10	12	Luzhou	241	28°51'01.9''	105°18'19.9''	Rice field ridge
Pop11	9	Yibin	317	28°45'15.4''	104°36'48.5''	Shrub at riverside
Pop12	5	Zigong	353	29°24'21.9''	104°49'01.4''	Grassland
Pop13	15	Jiangyou	687	31°58'06.8''	105°04'38.4''	Highway slope
Pop14	5	Jian’ge	611	32°13'25.0''	105°35'17.9''	Shrub at hillside
Pop15	18	Guangyuan	668	32°39'56.5''	105°56'21.4''	Shrub at hillside
Pop16	14	Daying	327	30°36'32.3''	105°13'59.6''	Shrub
Pop17	16	Banan	476	29°25'20.6''	106°34'37.5''	Forest edge at hillside
Pop18	11	Nanchuan	579	29°10'09.3''	107°06'44.1''	Shrub at hillside
Pop19	8	Dabai	455	28°32'51.8''	106°51'09.6''	Highway slope
Pop20	15	Zunyi	914	27°59'11.5''	106°52'25.1''	Coniferous edge
Pop21	12	Guiyang	1268	26°28'57.2''	106°27'35.8''	Bare rock
Pop22	6	Zhenning	1284	26°04'49.9''	105°46'55.9''	Glade
Pop23	6	Huangguoshu	946	25°58'43.6''	105°39'47.1''	Forest edge
Pop24	7	Yuxi	1721	24°11'53.2''	102°28'29.9''	Hillside
Total number of individuals	260					

### 3.2. DNA Extraction

Fresh young leaves from each sampled individual were collected and dried by desiccant (silicagel self indicator). Genomic DNA was extracted from the dried leaves using the Plant Genomic DNA kit (Tiangen^®^, Beijing, China) according to the manufacturer’s protocol. The quality and concentration of the DNA were determined by comparing the sample with known standards of lambda DNA on 0.8% (w/v) agarose gels. The isolated genomic DNA was diluted to 20 ng/μL and stored at −20 °C for PCR amplification.

### 3.3. Primer Selection and PCR-SRAP Amplification

SRAP primer sequences (Li and Qurios) [20] used in this study were synthesized by Shanghai Sangon Biological Engineering Technology & Service (Shanghai, China). For PCR amplification, the total volume of each PCR reaction was 20 μL, which contains 3 μL template DNA (20 ng/μL), 10 μL of Mix (10× reaction buffer, 2.0 mM Mg^2+^, 0.6 mM of each dNTPs, Tiangen), 0.8 μL primers (10 pmol/μL), 0.4 μL Golden DNA Polymerase (2.5 U/μL, Tiangen^®^) and 5 μL of ddH_2_O. Amplification was performed on a Peltier Thermal Cycler (DNA Engine^®^, Bio-Rad, Hercules, CA, USA) under the following conditions: 5 min at 94 °C, followed by 5 cycles at 94 °C for 1 min, 35 °C for 1 min, and 72 °C for 1 min, and then 35 cycles at 94 °C for 1 min, 50 °C for 1 min, and 72 °C for 1 min, extended at 72 °C for 10 min, then stored at 4 °C. The SRAP fragments were separated on 6% denatured polyacrylamide gels (acrylamide: bis-acrylamide 19:1, 1× TBE) and electrophoresis, later the gel were stained by AgNO_3_ solution and photographed by the Gel Doc XR system (Bio-Rad).

### 3.4. Data Analysis

For the statistical analysis, the SRAP banding patterns which could be unambiguously scored across all the sampled populations were recorded manually for band presence (1) or absence (0), each of them was treated as an independent character regardless of its intensity. The discriminatory power of different SRAP primers was evaluated by means of polymorphic information content (PIC) [53]. The resulting present/absent data matrix was analyzed using POPGENE32 v.1.31 [54]. Assuming Hardy-Weinberg equilibrium, the genetic diversity was evaluated with three parameters: the percentage of polymorphic loci (P), Nei’s [31] gene diversity (H) and Shannon’s Information Index of Diversity (I). The total gene diversity was given as (H_T_) which was divided into gene diversity within populations (H_S_) and the gene diversity among populations (D_ST_). These parameters were related according to the equation H_T_ = H_S_ + D_ST_. The genetic differentiation coefficient (G_ST_) was calculated as a ratio of D_ST_/H_T_, which was used to measure the population differentiation. The genetic distance (GD) among 24 populations were also computed using the same program [43]. Gene flow was calculated as Nm = 0.5(1 − G_ST_)/G_ST_ to estimate the level of gene drift among the populations [55]. Population structure of the 260 *M. sinensis* individuals was performed using STRUCTRE v2.3.4 software [56] with the ‘‘admixture model’’, burn-in period of 10,000 iterations and a run of 100,000 replications of Markov Chain Monte Carlo (MCMC) after burn in. For each run, 10 independent runs of STRUCTURE were performed with the number of clusters (K) varying from 1 to 11. Maximum likelihood and delta K (△K) values were used to determine the optimum number of subgroups [56,57]. For clustering analysis, the similarity coefficients were used to construct UPGMA (unweighted pair group method with arithmetic means) dendogram using SAHN (Sequential Agglomerative Hierarchical and Nested Clustering) module in the NTSYS-pc version2.10 software [58]. Genetic relationships among different *M. sinensis* populations were estimated using the Unweighted Pair-group Method with Arithmetic mean (UPGMA) cluster analysis based on the GD matrix. Analysis of molecular variance (AMOVA) was used to calculate variation among and within population using GenAlEx ver.6.41 [59].

## 4. Conclusions

SRAP markers were proved as useful tools in genetic diversity detection and population structure analysis. The 33 SRAP markers generated 688 bands with 646 as polymorphic bands. The average percentage of polymorphic bands (P), gene diversity (H), and Shannon’s diversity index (I) are 93.90%, 0.3377 and 0.5032 at species level respectively, indicating high level of genetic diversity. The mean genetic differentiation coefficient (Gst = 0.2326) estimated from 688 bands indicated that the larger genetic variation was found within populations which is consistent with the results calculated by the AMOVA analysis. In addition, there was a high frequency of gene flow (Nm = 1.6493) between populations, indicating there were more than one effective immigrant from one population into another at each generation. The population structure and phylogenetic analysis revealed five groups. Southwest China is located in one of the biodiversity hotspots of the world and the climate is variable. Additionally, *M. sinensis* is a cross-pollination plant, having complex genetic background and high heterozygosities. Hence, the genetic diversity and population structure analysis in the work reported here will facilitate genetic improvement and cultivar development with desired traits in further *M. sinensis* breeding programs.

## Supplementary Materials

Supplementary materials can be accessed at: http://www.mdpi.com/1420-3049/19/8/12881/s1.

## Supplementary File

Supplementary File 1
