# Supplementary material for: Genetic Variability and Population Structure of the Potential Bioenergy Crop Miscanthus sinensis (Poaceae) in Southwest China Based on SRAP Markers
Source: Molecules. 2014 Aug 21;19(8):12881–97. doi: 10.3390/molecules190812881 (PMC6271393; doi:10.3390/molecules190812881)

Supplementary Materials

Table S1. Overall proportion of membership of the 260 *M. sinensis* individuals in each of the 5 clusters.

Accession Code	1	2	3	4	5	Accession Code	1	2	3	4	5
0101	0.003	0.010	0.112	0.110	0.765	0908	0.005	0.003	0.006	0.927	0.060
0102	0.012	0.003	0.353	0.006	0.626	0909	0.006	0.005	0.020	0.957	0.011
0103	0.002	0.005	0.359	0.004	0.631	0910	0.002	0.003	0.008	0.983	0.005
0104	0.020	0.003	0.194	0.012	0.771	0912	0.003	0.011	0.005	0.977	0.004
0105	0.014	0.002	0.144	0.136	0.704	0914	0.003	0.004	0.003	0.988	0.003
0106	0.006	0.001	0.264	0.113	0.616	0915	0.007	0.022	0.002	0.965	0.004
0107	0.001	0.001	0.075	0.251	0.671	1001	0.009	0.001	0.002	0.981	0.007
0109	0.010	0.001	0.015	0.090	0.884	1003	0.065	0.003	0.004	0.906	0.023
0110	0.016	0.002	0.016	0.116	0.850	1004	0.016	0.001	0.041	0.933	0.009
0114	0.002	0.002	0.122	0.090	0.784	1005	0.007	0.003	0.040	0.948	0.002
0115	0.002	0.003	0.296	0.060	0.639	1006	0.002	0.018	0.002	0.971	0.007
0118	0.005	0.003	0.305	0.162	0.524	1007	0.009	0.001	0.010	0.976	0.004
0201	0.005	0.006	0.235	0.073	0.681	1008	0.007	0.001	0.003	0.986	0.003
0205	0.003	0.003	0.098	0.029	0.868	1010	0.003	0.006	0.003	0.975	0.013
0209	0.002	0.031	0.315	0.157	0.495	1102	0.024	0.002	0.139	0.833	0.002
0303	0.002	0.004	0.011	0.955	0.027	1103	0.012	0.068	0.100	0.814	0.005
0304	0.008	0.006	0.005	0.742	0.240	1107	0.010	0.032	0.002	0.954	0.002
0309	0.005	0.016	0.004	0.872	0.102	1109	0.110	0.005	0.004	0.880	0.002
0310	0.019	0.068	0.117	0.774	0.022	1110	0.003	0.005	0.027	0.961	0.004
0315	0.063	0.009	0.006	0.914	0.008	1112	0.034	0.022	0.010	0.924	0.011
0404	0.005	0.016	0.003	0.973	0.003	1204	0.008	0.005	0.010	0.974	0.003
0409	0.019	0.004	0.002	0.971	0.003	1206	0.048	0.003	0.039	0.901	0.009
0411	0.004	0.010	0.004	0.979	0.004	1207	0.019	0.003	0.024	0.926	0.027
0424	0.001	0.002	0.002	0.992	0.003	1209	0.017	0.001	0.146	0.835	0.002
0502	0.003	0.005	0.008	0.908	0.075	1212	0.008	0.019	0.018	0.944	0.012
0506	0.014	0.096	0.100	0.785	0.005	1302	0.007	0.028	0.041	0.913	0.011
0511	0.006	0.061	0.002	0.923	0.007	1303	0.115	0.005	0.013	0.860	0.007
0513	0.015	0.020	0.094	0.860	0.011	1306	0.033	0.040	0.002	0.898	0.027
0601	0.002	0.008	0.033	0.931	0.026	1307	0.008	0.016	0.003	0.971	0.003
0605	0.025	0.003	0.015	0.948	0.009	1310	0.005	0.005	0.005	0.983	0.002
0611	0.017	0.004	0.114	0.859	0.006	1312	0.033	0.010	0.007	0.931	0.019
0613	0.017	0.024	0.046	0.879	0.034	1404	0.036	0.017	0.019	0.900	0.028
0625	0.005	0.012	0.020	0.960	0.003	1407	0.003	0.007	0.016	0.965	0.010
0706	0.028	0.006	0.005	0.959	0.003	1410	0.035	0.003	0.078	0.827	0.056
0707	0.035	0.010	0.015	0.932	0.008	1412	0.006	0.003	0.005	0.982	0.003
0708	0.001	0.002	0.060	0.933	0.004	1502	0.069	0.014	0.292	0.622	0.004
0710	0.001	0.003	0.002	0.991	0.002	1504	0.003	0.007	0.023	0.827	0.140
0711	0.003	0.001	0.002	0.990	0.004	1506	0.039	0.002	0.016	0.938	0.005
0714	0.017	0.013	0.004	0.961	0.004	1507	0.056	0.047	0.101	0.686	0.110
0718	0.064	0.006	0.004	0.923	0.003	1509	0.022	0.008	0.205	0.760	0.005
0801	0.004	0.010	0.005	0.974	0.006	1510	0.018	0.003	0.108	0.864	0.007
0802	0.010	0.027	0.004	0.957	0.002	1512	0.002	0.002	0.023	0.967	0.006
0803	0.003	0.006	0.003	0.974	0.015	1514	0.009	0.013	0.020	0.764	0.195
0804	0.003	0.003	0.018	0.944	0.033	1601	0.003	0.121	0.009	0.861	0.006
0805	0.003	0.003	0.002	0.982	0.010	1602	0.109	0.002	0.009	0.854	0.026

Table S1. *Cont.*

Accession Code	1	2	3	4	5	Accession Code	1	2	3	4	5
0806	0.156	0.001	0.003	0.836	0.004	1603	0.004	0.032	0.003	0.950	0.010
0807	0.002	0.003	0.002	0.991	0.002	1605	0.002	0.051	0.003	0.943	0.001
0808	0.004	0.007	0.020	0.960	0.009	1606	0.017	0.022	0.026	0.924	0.012
0809	0.025	0.002	0.010	0.955	0.008	1609	0.004	0.072	0.005	0.917	0.003
0810	0.001	0.116	0.004	0.878	0.002	1701	0.016	0.016	0.007	0.950	0.010
0812	0.002	0.002	0.002	0.990	0.004	1704	0.010	0.010	0.008	0.912	0.060
0816	0.007	0.019	0.009	0.866	0.099	1706	0.057	0.114	0.028	0.788	0.013
0817	0.006	0.012	0.051	0.923	0.008	1707	0.002	0.051	0.004	0.923	0.020
0902	0.003	0.003	0.006	0.980	0.008	1708	0.016	0.003	0.005	0.969	0.006
0903	0.005	0.045	0.045	0.900	0.006	1709	0.021	0.003	0.004	0.966	0.005
0904	0.002	0.001	0.007	0.988	0.002	1802	0.065	0.028	0.018	0.826	0.063
0905	0.002	0.003	0.011	0.877	0.107	1805	0.041	0.039	0.021	0.887	0.012
0907	0.005	0.005	0.067	0.904	0.019	1806	0.003	0.011	0.012	0.967	0.008
1808	0.005	0.012	0.005	0.774	0.204	2714	0.004	0.003	0.377	0.002	0.614
1903	0.005	0.091	0.038	0.731	0.135	2715	0.005	0.003	0.301	0.020	0.671
1904	0.003	0.006	0.026	0.955	0.010	3102	0.002	0.001	0.008	0.001	0.988
1906	0.010	0.055	0.002	0.931	0.003	3116	0.001	0.004	0.004	0.001	0.990
1907	0.006	0.057	0.002	0.933	0.003	3201	0.006	0.002	0.012	0.005	0.975
1908	0.002	0.089	0.040	0.744	0.125	3202	0.045	0.040	0.006	0.001	0.907
2001	0.001	0.662	0.109	0.220	0.008	3204	0.003	0.001	0.103	0.001	0.891
2002	0.000	0.999	0.000	0.000	0.000	3205	0.010	0.006	0.004	0.012	0.968
2003	0.000	0.999	0.000	0.000	0.000	3206	0.004	0.059	0.004	0.032	0.901
2004	0.000	0.999	0.000	0.000	0.000	3207	0.003	0.098	0.076	0.008	0.816
2005	0.000	0.999	0.000	0.000	0.000	3208	0.005	0.009	0.004	0.004	0.979
2101	0.002	0.001	0.995	0.001	0.001	3209	0.003	0.001	0.272	0.002	0.722
2102	0.017	0.004	0.956	0.009	0.014	3210	0.001	0.002	0.061	0.006	0.930
2103	0.002	0.002	0.993	0.001	0.002	3211	0.009	0.022	0.008	0.077	0.885
2104	0.003	0.002	0.991	0.002	0.002	3212	0.007	0.001	0.009	0.002	0.980
2105	0.005	0.005	0.966	0.005	0.018	3213	0.002	0.004	0.012	0.003	0.979
2106	0.002	0.007	0.962	0.010	0.019	3215	0.003	0.003	0.015	0.214	0.765
2107	0.005	0.024	0.926	0.008	0.037	3216	0.005	0.012	0.002	0.002	0.978
2108	0.006	0.015	0.948	0.009	0.022	3302	0.032	0.002	0.162	0.786	0.019
2109	0.003	0.004	0.964	0.010	0.019	3306	0.005	0.025	0.007	0.532	0.431
2110	0.003	0.003	0.957	0.026	0.010	3308	0.002	0.046	0.005	0.520	0.426
2111	0.002	0.001	0.993	0.003	0.002	3309	0.110	0.011	0.004	0.616	0.258
2112	0.003	0.002	0.947	0.043	0.004	3311	0.007	0.021	0.002	0.751	0.219
2203	0.001	0.001	0.991	0.003	0.003	3312	0.007	0.159	0.003	0.577	0.254
2204	0.002	0.002	0.980	0.008	0.008	3313	0.002	0.003	0.002	0.876	0.117
2205	0.005	0.001	0.757	0.004	0.233	3314	0.001	0.003	0.003	0.955	0.037
2302	0.084	0.001	0.326	0.576	0.013	3315	0.040	0.030	0.005	0.899	0.027
2303	0.006	0.005	0.319	0.668	0.002	3316	0.002	0.007	0.004	0.782	0.205
2304	0.031	0.001	0.241	0.724	0.003	3317	0.026	0.002	0.003	0.745	0.225
2305	0.053	0.003	0.206	0.736	0.002	3601	0.117	0.015	0.003	0.628	0.236
2306	0.063	0.001	0.318	0.616	0.001	3602	0.104	0.008	0.006	0.630	0.252
2401	0.002	0.001	0.483	0.084	0.429	3604	0.027	0.119	0.005	0.631	0.217
2402	0.003	0.002	0.484	0.241	0.269	3605	0.125	0.005	0.003	0.859	0.007

Table S1. *Cont.*

Accession Code	1	2	3	4	5	Accession Code	1	2	3	4	5
2403	0.006	0.002	0.556	0.183	0.255	3606	0.021	0.072	0.004	0.609	0.295
2404	0.016	0.010	0.812	0.053	0.109	3607	0.003	0.049	0.008	0.796	0.144
2405	0.041	0.001	0.885	0.005	0.067	3608	0.005	0.002	0.004	0.973	0.016
2406	0.001	0.001	0.841	0.003	0.154	3609	0.027	0.001	0.017	0.931	0.023
2407	0.001	0.002	0.862	0.003	0.132	3701	0.003	0.027	0.003	0.125	0.843
2408	0.058	0.002	0.923	0.003	0.013	3702	0.024	0.028	0.047	0.188	0.713
2409	0.011	0.006	0.901	0.011	0.072	3703	0.002	0.004	0.027	0.038	0.929
2410	0.106	0.034	0.830	0.006	0.024	3704	0.006	0.009	0.003	0.115	0.868
2501	0.005	0.012	0.864	0.002	0.117	3705	0.012	0.004	0.045	0.015	0.924
2502	0.009	0.018	0.966	0.002	0.005	3706	0.002	0.003	0.012	0.036	0.946
2503	0.026	0.002	0.864	0.095	0.013	3707	0.008	0.028	0.113	0.056	0.794
2505	0.001	0.023	0.912	0.021	0.043	3708	0.004	0.005	0.074	0.002	0.915
2506	0.007	0.002	0.954	0.009	0.028	3709	0.010	0.014	0.032	0.014	0.930
2601	0.005	0.008	0.277	0.708	0.002	3710	0.009	0.014	0.005	0.057	0.916
2604	0.012	0.008	0.491	0.487	0.002	3711	0.003	0.007	0.006	0.009	0.976
2606	0.004	0.005	0.293	0.697	0.002	3712	0.013	0.001	0.025	0.012	0.949
2701	0.002	0.002	0.396	0.003	0.597	3713	0.010	0.002	0.009	0.204	0.774
2702	0.021	0.020	0.433	0.005	0.521	3714	0.026	0.002	0.002	0.025	0.945
2703	0.005	0.001	0.363	0.003	0.628	3715	0.008	0.007	0.002	0.085	0.899
2704	0.004	0.034	0.445	0.003	0.514	3802	0.062	0.004	0.003	0.908	0.023
2705	0.035	0.007	0.507	0.012	0.439	3804	0.041	0.006	0.002	0.720	0.231
2706	0.003	0.004	0.425	0.004	0.563	3806	0.016	0.104	0.046	0.772	0.061
2707	0.021	0.006	0.498	0.049	0.426	3807	0.056	0.002	0.004	0.911	0.028
2709	0.004	0.006	0.398	0.062	0.530	3808	0.037	0.002	0.001	0.720	0.240
2710	0.003	0.072	0.443	0.023	0.459	3809	0.054	0.029	0.003	0.578	0.336
2711	0.011	0.001	0.458	0.002	0.527	3810	0.065	0.112	0.001	0.494	0.328
2712	0.009	0.003	0.343	0.001	0.644	3903	0.123	0.007	0.003	0.788	0.080
2713	0.003	0.008	0.432	0.016	0.542	3904	0.011	0.031	0.101	0.779	0.079
3905	0.005	0.046	0.002	0.564	0.382	4109	0.012	0.002	0.003	0.771	0.212
3906	0.041	0.006	0.010	0.642	0.301	4111	0.007	0.020	0.003	0.740	0.231
3907	0.070	0.001	0.011	0.786	0.132	4702	0.551	0.001	0.037	0.308	0.103
4001	0.027	0.002	0.008	0.727	0.235	4705	0.994	0.003	0.001	0.001	0.001
4003	0.034	0.035	0.005	0.655	0.271	4706	0.996	0.001	0.000	0.001	0.001
4004	0.081	0.007	0.003	0.772	0.137	4710	0.998	0.001	0.000	0.000	0.000
4005	0.023	0.001	0.009	0.662	0.305	4713	0.998	0.001	0.000	0.001	0.000
4006	0.125	0.007	0.003	0.717	0.148	4714	0.998	0.001	0.000	0.000	0.001
4007	0.074	0.001	0.002	0.618	0.306	4715	0.994	0.002	0.001	0.001	0.001

Note: The accession code 0101–0209 were contained Pop1; the accession code 0303–0513 were contained Pop2; the accession code 0601–0718 were contained Pop3; the accession code 0801–0817 were contained Pop4; the accession code 0902–0915 were contained Pop5; the accession code 1001–1010 were contained Pop6; the accession code 1102–1212 were contained Pop7; the accession code 1302–1412 were contained Pop8; the accession code 1502–1514 were contained Pop9; the accession code 1601–1709 were contained Pop10; the accession code 1802–1908 were contained Pop11; the accession code 2001–2005 were contained Pop12; the accession code 2101–2205 were contained Pop13; the accession code 2302–2306 were contained Pop14; the accession code 2401–2606 were contained Pop15; the accession code 2701–2715 were contained Pop16; the accession code 3102–3216 were contained Pop17; the accession code 3302–3317 were contained Pop18; the accession code 3601–3609 were contained Pop19; the accession code 3701–3715 were contained Pop20; the accession code 3802–3907 were contained Pop21; the accession code 4001–4007 were contained Pop22; the accession code 4102–4111 were contained Pop23; the accession code 4702–4715 were contained Pop24.

Table S2. Nei's unbiased measures of genetic distance.

Pop	1	2	3	4	5	6	7	8	9	10	11	12	13	14	15	16	17	18	19	20	21	22	23
1																							
2	0.077																						
3	0.078	0.028																					
4	0.085	0.033	0.030																				
5	0.085	0.046	0.042	0.047																			
6	0.105	0.055	0.056	0.058	0.061																		
7	0.086	0.044	0.038	0.049	0.048	0.049																	
8	0.086	0.044	0.044	0.048	0.054	0.064	0.040																
9	0.083	0.049	0.050	0.049	0.057	0.071	0.045	0.040															
10	0.087	0.044	0.054	0.052	0.057	0.066	0.045	0.052	0.051														
11	0.096	0.062	0.066	0.066	0.060	0.078	0.058	0.057	0.064	0.050													
12	0.228	0.166	0.181	0.188	0.187	0.190	0.174	0.172	0.191	0.164	0.171												
13	0.059	0.093	0.089	0.098	0.100	0.125	0.089	0.098	0.086	0.101	0.110	0.246											
14	0.138	0.107	0.110	0.109	0.119	0.120	0.097	0.121	0.119	0.107	0.118	0.241	0.126										
15	0.059	0.080	0.079	0.085	0.089	0.110	0.079	0.085	0.074	0.086	0.095	0.230	0.034	0.105									
16	0.053	0.079	0.087	0.088	0.094	0.111	0.084	0.092	0.084	0.085	0.099	0.227	0.050	0.131	0.047								
17	0.054	0.080	0.090	0.093	0.096	0.119	0.086	0.090	0.082	0.087	0.097	0.225	0.066	0.152	0.058	0.038							
18	0.075	0.055	0.049	0.051	0.057	0.076	0.059	0.059	0.064	0.060	0.064	0.188	0.095	0.094	0.076	0.080	0.073						
19	0.093	0.061	0.060	0.065	0.071	0.075	0.063	0.066	0.076	0.064	0.069	0.185	0.111	0.109	0.098	0.099	0.098	0.053					
20	0.057	0.079	0.083	0.083	0.087	0.105	0.087	0.087	0.078	0.088	0.094	0.214	0.074	0.143	0.068	0.052	0.040	0.071	0.090				
21	0.087	0.066	0.064	0.067	0.076	0.082	0.065	0.074	0.076	0.068	0.083	0.199	0.100	0.114	0.091	0.086	0.081	0.048	0.058	0.072			
22	0.102	0.075	0.079	0.081	0.087	0.091	0.080	0.081	0.096	0.084	0.090	0.208	0.121	0.128	0.111	0.108	0.102	0.070	0.075	0.087	0.054		
23	0.113	0.087	0.091	0.092	0.094	0.101	0.099	0.091	0.110	0.088	0.097	0.208	0.135	0.141	0.125	0.122	0.120	0.088	0.081	0.108	0.074	0.067	
24	0.188	0.156	0.154	0.162	0.174	0.162	0.140	0.148	0.164	0.163	0.172	0.292	0.188	0.194	0.176	0.176	0.178	0.163	0.147	0.178	0.151	0.159	0.176

Figure S1. Dendrogram of 260 *M. sinensis* individuals based on GS data by UPGMA cluster analysis.

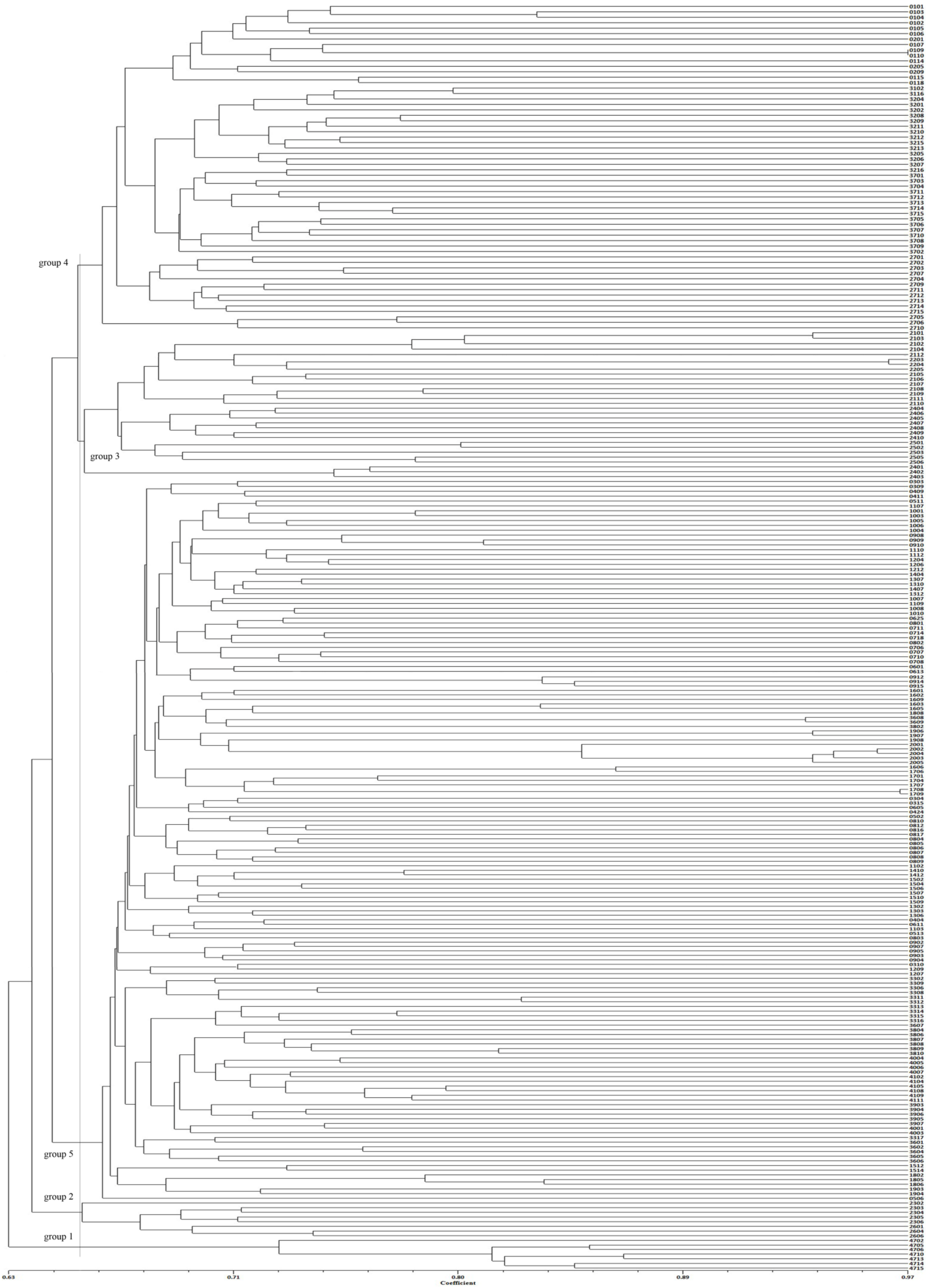

Supplement: Supplementary File 1 [file molecules-19-12881-s001.pdf]
